# Telemanagement of Home-Isolated COVID-19 Patients Using Oxygen Therapy With Noninvasive Positive Pressure Ventilation and Physical Therapy Techniques: Randomized Clinical Trial

**DOI:** 10.2196/23446

**Published:** 2021-04-28

**Authors:** Aya Sedky Adly, Mahmoud Sedky Adly, Afnan Sedky Adly

**Affiliations:** 1 Faculty of Computers and Artificial Intelligence Helwan University Cairo Egypt; 2 Faculty of Engineering and Technology Badr University in Cairo (BUC) Cairo Egypt; 3 Faculty of Oral and Dental Medicine Cairo University Cairo Egypt; 4 Royal College of Surgeons of Edinburgh Scotland United Kingdom; 5 Faculty of Physical Therapy Cardiovascular-Respiratory Disorders and Geriatrics, Laser Applications in Physical Medicine Cairo University Cairo Egypt; 6 Faculty of Physical Therapy Internal Medicine Beni-Suef University Beni-Suef Egypt

**Keywords:** telemedicine, oxygen therapy, noninvasive positive airway pressure, BiPAP, osteopathic medicine, physical therapy, SARS-CoV-2, COVID-19, teletherapy, telemanagement

## Abstract

**Background:**

With the growing stress on hospitals caused by the COVID-19 pandemic, the need for home-based solutions has become a necessity to support these overwhelmed hospitals.

**Objective:**

The goal of this study was to compare two nonpharmacological respiratory treatment methods for home-isolated COVID-19 patients using a newly developed telemanagement health care system.

**Methods:**

In this single-blinded randomized clinical trial, 60 patients with stage 1 pneumonia caused by SARS-CoV-2 infection were treated. Group A (n=30) received oxygen therapy with bilevel positive airway pressure (BiPAP) ventilation, and Group B (n=30) received osteopathic manipulative respiratory and physical therapy techniques. Arterial blood gases of PaO_2_ and PaCO_2_, pH, vital signs (ie, temperature, respiratory rate, oxygen saturation, heart rate, and blood pressure), and chest computed tomography scans were used for follow-up and for assessment of the course and duration of recovery.

**Results:**

Analysis of the results showed a significant difference between the two groups (*P*<.05), with Group A showing shorter recovery periods than Group B (mean 14.9, SD 1.7 days, and mean 23.9, SD 2.3 days, respectively). Significant differences were also observed between baseline and final readings in all of the outcome measures in both groups (*P*<.05). Regarding posttreatment satisfaction with our proposed telemanagement health care system, positive responses were given by most of the patients in both groups.

**Conclusions:**

It was found that home-based oxygen therapy with BiPAP can be a more effective prophylactic treatment approach than osteopathic manipulative respiratory and physical therapy techniques, as it can impede exacerbation of early-stage COVID-19 pneumonia. Telemanagement health care systems are promising methods to help in the pandemic-related shortage of hospital beds, as they showed reasonable effectiveness and reliability in the monitoring and management of patients with early-stage COVID-19 pneumonia.

**Trial Registration:**

ClinicalTrials.gov NCT04368923; https://clinicaltrials.gov/ct2/show/NCT04368923

## Introduction

COVID-19 has become a global pandemic that has had a gross dramatic impact on hospitals and health care systems worldwide [1-3]. Therefore, home isolation may become the only available option to stop the spread in most countries [4-6]. A worldwide shortage of medical devices, protective equipment, and pharmacological treatment with growing stress on hospital resources have led to an obvious drop in the performance of the majority of frontline health care workers [7-9]. Therefore, introducing telemanagement approaches has become a necessity for coping with the exponential increase in the number of people infected with SARS-CoV-2 [10-12].

Home care isolation can be considered for COVID-19 patients with mild illness when inpatient isolation is unavailable. However, advice and precautions regarding respiratory hygiene, environmental ventilation, hand hygiene, shared space confinement, and optimal nutritional intake should be followed as a part of the management process [13,14].

Current evidence suggests that the application of noninvasive bilevel positive airway pressure (BiPAP) can reduce pulmonary complications and raise pulmonary oxygen pressure by activation of collapsing alveoli and reduction of the degree of shunt [15]. However, noninvasive positive pressure ventilation is usually considered the last line of treatment in the initial management of hospitalized COVID-19 patients, as it is considered an aerosol-generating procedure that can increase the risk of infection in a hospital setting [16,17]. Yet, there is an apparent lack of research on its impact on COVID-19 patients, despite evidence from several previous studies that confirm its beneficial effects in different types of pneumonia.

COVID-19 disease progression and complications have been found to be unpredictable. Patients’ deterioration has been commonly reported to be a result of pulmonary edema due to interstitial fluid accumulation from pulmonary capillary leakage, cytokine storms, and microvascular thrombosis. Pulmonary edema is characterized by acute onset and rapid progression [18]. Noninvasive BiPAP may prevent this deterioration if applied at an early stage by impeding the consequent pulmonary edema via positive pressure [19].

Recent studies indicate that osteopathic manipulative respiratory and physical therapy techniques can improve pulmonary function in both chronic and acute pulmonary conditions [20]. These techniques are directed toward the respiratory musculoskeletal components inducing thoracic pressure changes, which are essential for effective respiratory processes. Additionally, these techniques have enormous potential in the alleviation of pulmonary disease complications through increasing mobility of chest wall muscles and the diaphragm [21].

Osteopathic manipulative respiratory and physical therapy techniques have an important advantage of being low cost with the ability to be modified for home or self-application, which can reduce the risk of infection and overcome the shortage in pharmacological treatments and medical devices [22].

In the presence of COVID-19, telepractice has transformed physical therapy, as communication-based platforms beyond telerehabilitation, telemedicine, telemanipulation, and telehealth are utilized to advance remote access to therapy [23].

Recent evidence supported the use of self-directed web-based physical therapy over traditional outpatient physical therapy. Moreover, web-based physical therapy at home has gained interest owing to its interactive nature, which offers a more formal structure and direction for the patients [24]. Consequently, self-directed web-based physical therapy with remote supervision can be considered as an effective solution to offset the risks associated with infection among COVID-19 patients without compromising outcomes [24-26].

Noninvasive ventilation (NIV) has been utilized as an adjunct to physical therapy in patients with respiratory diseases. Several studies have demonstrated the positive effects of NIV and supplemental oxygen as adjuncts to physical therapy [27]. NIV was found to unload the ventilatory muscles and reverse the neural drive of fatigue through reducing the amount of necessary patient effort as well as reducing the muscle load during an assisted interactive breath [28]. Thus, investigation of these two treatment modalities among COVID-19 patients would be a great benefit.

Evidence has reported that real-time telemedicine in patients with acute respiratory infections showed similar rates of clinical management when compared to traditionally treated patients. Furthermore, the frequency of follow-up visits in real-time telemedicine was higher than in traditional follow-up visits [29].

Preliminary regulations have been issued regarding the control of infection, diagnosis, and monitoring of COVID-19 patients, while there is limited and unclear guidance on the effect of starting care management of these patients from an early stage [30-33]. The divergence of results in the majority of research studies on this topic and the need for high-quality randomized clinical trials that follow the recommended standards of reporting were the reasons for implementing this study.

Advances in information and communication technologies (ICTs) have turned the world into a connected village. Remarkable challenges have been addressed by ICTs in various health care sectors [34-36].

Thus, the aim of this study was to assess and compare oxygen therapy combined with noninvasive positive pressure ventilation with osteopathic manipulative respiratory and physical therapy techniques using a telemanagement health care system, which was applied to home-isolated COVID-19 patients. We also assessed patient satisfaction with the COVID-19 telemanagement system.

## Methods

### Overview

**Figure 1:**
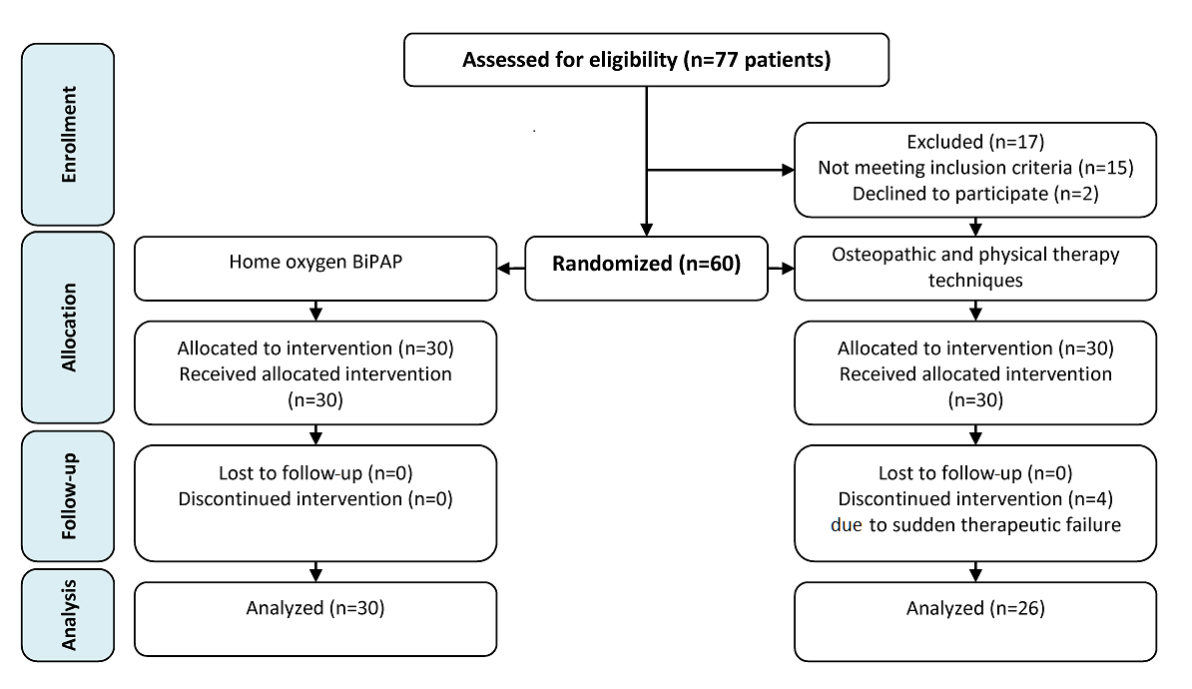
CONSORT flow diagram. BiPAP: bilevel positive airway pressure.

This single-blinded, parallel-group, randomized clinical trial was approved by the Research Ethics Committee of Cairo University and was registered at ClinicalTrials.gov (NCT04368923). The study was performed in accordance with the ethical standards of the Declaration of Helsinki and followed CONSORT guidelines for conducting randomized trials (see Figure 1).

Patient recruitment was realized through social media using the snowball subject recruitment technique. Initially, invitations were targeted to patients who met the study’s eligibility criteria through the health professionals’ social media networks. Then, those patients were requested to distribute invitations through their individual social media networks; no restrictions were made on what social media platform they should use. The participants were obligated to send the required data online in order to be assessed for eligibility.

A total of 60 patients were randomized into two groups in a 1:1 allocation ratio using the computer-generated randomization software StatsDirect, version 2.7.7 (StatsDirect Ltd); the allocation was done by a blinded and independent coworker to ensure that the randomization process was totally concealed. This study was conducted with a sample of home-isolated patients. Informed consent was obtained from all patients who were included in this study.

The inclusion criteria for patients were as follows:

- American Society of Anesthesiologists Class I patient before the onset of COVID-19
- History of close contact to a confirmed positive COVID-19 case as defined by the national guidelines for public health units [37]
- SARS-CoV-2 infection determined by chest computed tomography (CT) scan showing typical ground-glass abnormalities with the onset of two or more clinical symptoms and the patient condition classified as stage 1 pneumonia according to Pan et al [38]
- Patient indicated for home isolation.

Patients were excluded if they demonstrated the following characteristics:

- Not being consistent with home-isolation regimen, including nutritional supplementation, proper hygiene, and room aeration
- Inability to deal with the telemanagement system provided
- Therapeutic failure during the study, which is characterized by severe respiratory distress with respiratory rate that is more than or equal to 30 breaths per minute, oxygen saturation less than or equal to 93% in room air, and a requirement of intubation or mechanical ventilation [39].

Patients were divided equally into two groups, with 30 patients in each group. The first group received oxygen therapy with BiPAP ventilation (Group A), and the second group received osteopathic manipulative respiratory and physical therapy techniques (Group B). All patients received the same nutritional supplementation, including multivitamins and adequate supportive diets.

Regarding Group A, an oxygen concentrator with BiPAP was given using an AirFit F30 face mask (ResMed) with inspiratory positive airway pressure/expiratory positive airway pressure of 15/3 cm H_2_O and 5 L/min oxygen flow by an oxygen flow meter, which was done for 4 hours per day [40]. Procedures for oxygen administration were consistent across all patients, clarified in detail, and accomplished via a teleconference for each patient that was supervised by an expert respiratory physiotherapist.

Group B received osteopathic manipulative respiratory and physical therapy techniques in the form of the following:

- Prone reverse Trendelenburg positioning for 4 hours/day [41,42]
- Cephalic traction with approximate duration of 1 minute/day [43]
- Muscle energy technique for scalene muscles 5 times/day [43]
- Rib raising technique (5 cycles for each rib group with a total of 15 cycles and approximately 5 minutes’ duration) [44]
- Suboccipital area intermittent rhythmic pressure (according to each patient sensitivity) [45]
- Osteopathic lymphatic thoracic pump techniques with respiratory assist for 4 minutes [46]
- Pedal lymphatic pump for 1 minute [47]
- Thoracic inlet myofascial release for 1 minute [48]
- Diaphragmatic doming for 3 to 5 sequential respiratory cycles [49-52].

A real-time videoconference was established between the patient and the physiotherapist for training, directing, and supervising the patient during self-application. All techniques were given on a daily basis. The total duration of each therapeutic session was approximately 4 hours and 30 minutes.

In both groups, therapy ended after the assessment of all the outcome values, making sure that all of them fell within the normal range and continued to be stable for three consecutive outcomes without any clinical symptoms, which indicated recovery.

Evaluation procedures included the following:

- Recording of arterial blood gases for both oxygen (PaO_2_) and carbon dioxide (PaCO_2_) in addition to pH for each patient in both groups every 48 hours [53]
- Telemonitoring of vital signs (ie, temperature, respiratory rate, oxygen saturation, heart rate, and blood pressure) for each patient in both groups every 24 hours [54]
- Pretreatment and 14 days’ posttreatment chest CT scans.

All evaluation procedures were recorded, monitored, and analyzed with an application developed by the authors, which was used for management of the patients’ data and tracking of their therapeutic progress. Continuous online support with comprehensive supervision was done via videoconferencing by expert respiratory physiotherapists whenever requested by the patient.

The primary outcome measures for this study were the times to reach normal levels of both PaO_2_ and PaCO_2_ in addition to pH, which were assessed every 48 hours. Secondary outcome measures were temperature, respiratory rate, oxygen saturation, heart rate, and blood pressure, which were evaluated every 24 hours.

The participants were provided with wearable devices that have been used for telemonitoring of the required vital signs. The devices were capable of transmitting vital signs data via Bluetooth connection to the authorized gateway (ie, mobile, tablet, or any other gateway).

Collection and reporting of the primary outcome measures were done by three laboratory technicians who were blinded to the groups of study; they were then analyzed via the system. The technicians were assigned to take the samples from patients’ homes for analysis. Interrater reliability was assessed using the intraclass correlation coefficient, which was 0.97; intrarater reliability was assessed using the Pearson correlation coefficient and was found to be 0.99.

Secondary outcome measures were collected and reported by the patient himself or herself, and the evaluation of these readings was done by a single clinician who was also blinded to the groups under study. All patients were instructed to be observed for another 14 days after ending therapy; chest CT scans were then performed.

### Method for Telemanagement of COVID-19 Patients

A telemanagement system was developed by the authors, which followed the Health Level Seven Version 3 Standard in order to simplify its integration and facilitate receiving and/or retrieving information from other sources and applications, as well as to enable the usage of Internet of Things. This system was able to support live transmission of the vital signs data and allowed for incorporation of different medical sensors through wireless connections. The patient was able to access the system via mobile phone, tablet, or any web platform.

The major components of the platform included (1) a thorough monitoring plan, (2) the patient-specific interventions, (3) definitions of alarms and indicators, (4) the user-environment configurations of the therapist’s device, and (5) the user-environment configurations of the patient’s device.

Additionally, the system was able to provide an efficient, flexible, scalable integrated solution that incorporates artificial intelligence to provide support in planning, predicting, and decision making. The platform was specifically designed for managing and providing teletherapeutic services to home-isolated COVID-19 patients. It also covered the services of therapists and managing staff while coordinating the work of all the involved professionals. The system was also able to allow the patients to receive an individualized therapeutic program. It also allowed the therapists to set up a plan and thresholds that could be personalized in accordance with each patient’s profile.

In addition, it provided the option to create combined alerts via several variables. The system provided the patient with step-by-step written instructions as well as precautions about each procedure, along with a video that explained how to perform the procedures. Another advantage of this system was the auto-reminder feature to help patients with sending their data on time.

After outcome data were collected for each patient, the results were sent immediately to a server so they would be available for analysis. The system included a decision support option, which provided tailored feedback for each patient. Depending on the progress of the outcome variables, the system provided an alerting option when certain outcomes were reached or when patient performance required in-person counseling. At the end of the therapy, the system enabled each patient to answer posttreatment questions to assess patient satisfaction with the quality of this novel telemanagement service.

The decision support engine entailed workflow management routines that were used for coordinating reception of the inputs and managing their interactions, in addition to handling decision support outputs by means of tasks or actions.

The alert messages were sent to the intended devices with features that could be adjusted, such as color-coded displays, preferred choices for creating alerts, and interface personalization options. The system presented the information in meaningful medical-related ways, where it first presented the alerts followed by their related information, which was gradually presented in a more detailed manner.

With the goal of adapting the designed platform in line with the specific needs of COVID-19 patients, we conducted consecutive meetings with the patients and the therapists. The platform took into consideration the normal values of the outcome measures according to their follow-up schedule. Furthermore, we incorporated a group of educational elements for improving patients’ knowledge of COVID-19, which was strengthened via interactive materials.

Analysis began after the reception of data, or was periodically scheduled, in accordance with decision support requirements. The input-module functions were dependent on the routines of feature extraction for the characterization of the data patterns.

Data sets were generated through the collection of questionnaires and clinical measurements; these measurements were assessed as being a function of time in order to detect sudden deviations, which can indicate health deterioration. The feature extraction routines for the distribution analysis and thresholding were implemented as separated modules; processed data were then retrieved and the notable clinical data patterns were fed back into a decision support engine.

For proper identification of the significant downward or upward values over the analysis window period, an identification analysis routine was used. In addition, a threshold analysis routine was used for comparing the values to the adaptive thresholds. The data in the outer ranges were defined through the confidence intervals, and any detected deviation was flagged as being an abnormal measurement. Furthermore, we designed the system to have the ability to predict the probability that a future patient would experience therapeutic failure using deep learning; however, a sufficient training data set is yet to be obtained in order for the system to provide accurate results.

Deep learning was based on feed-forward multilayer artificial neural networks that used back-propagation processes for updating the weights among the hidden layers and the output; a back-propagation of the resulting error was then applied. For the predictions, the mean square error, as well as the *R*^2^ error, were calculated.

### Statistical Analysis

The sample size was calculated with a power of 80% and level of significance of 5% (α=.05). The analysis was based on post hoc power analyses utilizing G*Power software, version 3 (Heinrich-Heine-Universität Düsseldorf) [55], accounting for 15% missing data, and based on the minimal meaningful effect size. This analysis showed that the sample size was adequate. Differences between the two groups were assessed by means and standard deviations. Comparison between the two groups was done using Student *t* test. Nominal data were summarized as frequencies and percentages. All data were statistically analyzed by an independent statistician, who was blinded to the interventions, using SPSS statistical software, version 20 (IBM Corp).

## Results

A total of 60 patients (22 males [37%] and 38 females [63%]) were included in this study. Their ages ranged from 21 to 40 years with a mean age of 31.6 (SD 5.8) years. The characteristics that presented most frequently were fever (56/60, 93%) and dyspnea (55/60, 92%), as shown in Table 1.

**Table 1:** Demographics and baseline characteristics of patients.

Characteristic		Total (N=60)	Group A (n=30)	Group B (n=30)
Age (years), mean (SD)		31.6 (5.8)	32.2 (5.4)	30.9 (6.2)
**Gender, n (%)**				
	Male	22 (37)	10 (33)	12 (40)
	Female	38 (63)	20 (67)	18 (60)
**Clinical symptom, n (%)^a^**				
	Fever	56 (93)	27 (90)	29 (97)
	Tachypnea	51 (85)	29 (97)	22 (73)
	Tachycardia	29 (48)	17 (57)	12 (40)
	Hypertension	19 (32)	11 (37)	8 (27)
	Dyspnea	55 (92)	27 (90)	28 (93)
	Cough	43 (72)	17 (57)	26 (87)
	Chest tightness	12 (20)	5 (17)	7 (23)

^a^Percentages in this category add up to greater than 100% because patients could report multiple symptoms.

**Figure 2:**
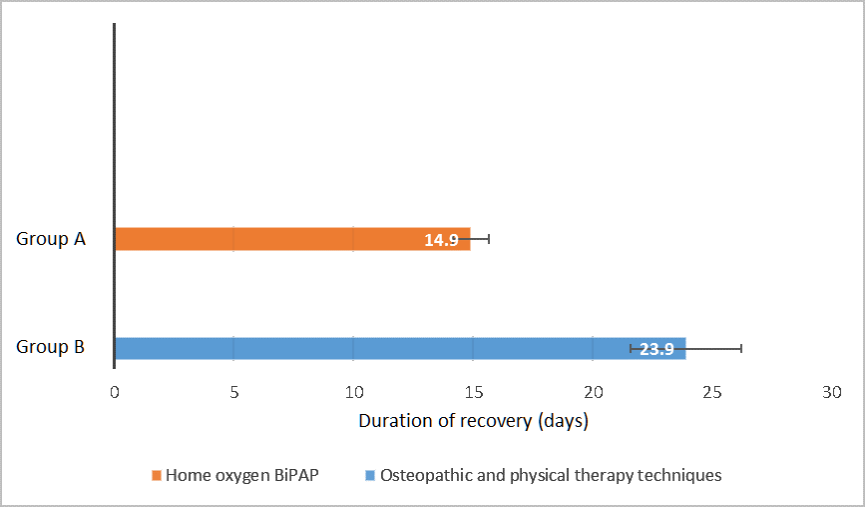
Number of days needed for Group A and Group B to recover. Values and whiskers on bars are means and SDs, respectively. BiPAP: bilevel positive airway pressure.

**Figure 3:**
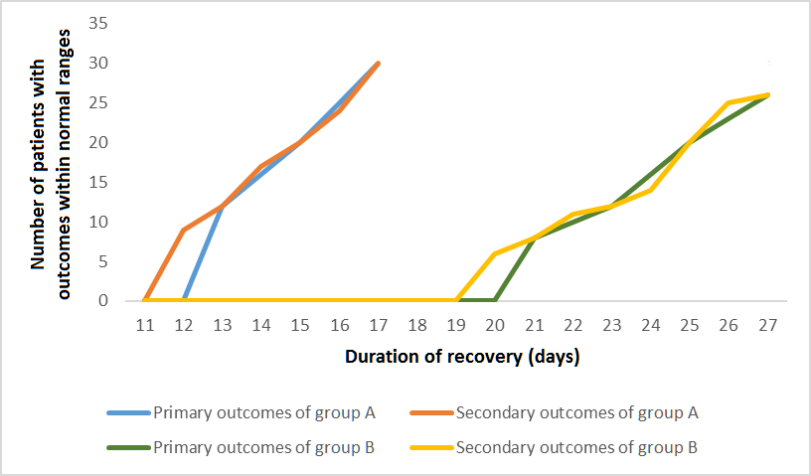
Patient outcome recovery rates.

A significant difference was observed between the two groups regarding the mean number of days needed for recovery (*P*<.05), with Group A showing a lower recovery period than Group B (mean 14.9, SD 1.7 days, and mean 23.9, SD 2.3 days, respectively), as shown in Figures 2 and 3. The unpaired *t* test (*df*=58) value was 16.55 with a mean difference of –9.056 between Group A and Group B.

A total of 4 patients out of 30 (13%) from Group B were excluded because of sudden therapeutic failure during the course of osteopathic manipulative respiratory and physical therapy techniques, as these patients required hospitalization and/or intubation.

All patients who were included in the analysis underwent chest CT scans two times, one before starting therapy and the other 14 days after ending therapy. Early-stage COVID-19 pneumonia mainly appeared as minor subpleural, bilateral, or, less commonly, unilateral ground-glass opacities in the lower lobes.

A total of 51 of the 60 patients (85%) had bilateral lung pneumonia, while 9 of the 60 patients (15%) had unilateral lung involvement. The unilateral lung involvement cases were comprised of 6 patients who had right lung involvement and 3 patients who had left lung involvement.

A total of 2 weeks after ending therapy, the CT scans showed complete resolution in Group A patients; however, in Group B patients, the lesions were mostly absorbed compared with images from before therapy in 23 out of 30 patients (77%), while 3 patients (10%) showed no worsening compared to the previous CT scan results. The 4 patients who were excluded from the study due to therapeutic failure were instructed to be admitted to the hospital.

The data sets from the study were based on the daily reports. These reports included analysis of arterial blood gases and vital signs. The data set consisted of 1735 daily records. After filtering the data set by removing the records of the excluded patients, the final analyzed data set included 1686 records. The parameters of the submitted records for each attribute are shown in Table 2.

**Table 2:** Parameters of the submitted records.

Attribute	Duration	Total submitted records for Group A, n	Total submitted records for Group B, n
PaO_2_	Every 48 hours	238	324
PaCO_2_	Every 48 hours	238	324
pH	Every 48 hours	238	324
Temperature	Every 24 hours	476	648
Respiratory rate	Every 24 hours	476	648
Oxygen saturation	Every 24 hours	476	648
Heart rate	Every 24 hours	476	648
Blood pressure	Every 24 hours	476	648

Our system predictions showed an *R*^2^ of 0.965 with a mean square error of 0.27, which means that more than 96% of the variations were predicted.

The posttreatment patient satisfaction questions focused on a group of technical aspects—simplicity, effectiveness, acceptability, usability, reliability, and level of confidence—as well as the system’s ability to assess a patient’s status remotely. The results of the satisfaction questions were very promising and most of the patients responded positively to the questions, as shown in Table 3.

**Table 3:** Posttreatment patient satisfaction questions for the telemanagement system.

Question	Response (n=56), n (%)^a^			
	Yes	No	No answer	
Were the telemanagement procedures simple?	41 (73)	12 (21)	3 (5)	
Were the telemanagement treatment procedures useful?	54 (96)	0 (0)	2 (4)	
Were the telemanagement procedures well tolerated?	56 (100)	0 (0)	0 (0)	
Were educational elements and interactive materials useful?	56 (100)	0 (0)	0 (0)	
Did you regret using this telemanagement system and, instead, prefer admission to the hospital?	1 (2)	52 (93)	3 (5)	
Did the auto-reminder option in this system help you in sending data on time?	55 (98)	0 (0)	1 (2)	
Do you think that the time spent by therapists with you was adequate?	38 (68)	13 (23)	5 (9)	
Do you think that this telemanagement system was consistent?	49 (88)	2 (4)	5 (9)	
Do you think that this telemanagement system is an acceptable way to receive treatment services?	56 (100)	0 (0)	0 (0)	
Overall, are you satisfied with the quality of the services provided by this telemanagement system?	55 (98)	1 (2)	0 (0)	
Would you recommend this telemanagement system to anyone?	51 (91)	1 (2)	4 (7)	
Any comments? (Comments added to a free-text field are shown below) I regret not being admitted to the hospital as I think that I would not have transmitted the infection to my wife. This telemanagement system saved efforts, costs, and time. I think that the time spent with the therapist needs to be more frequent. The system could be more helpful if it would send periodic information often about my status by informing me if I am getting better or worse.	4 (7)	48 (86)	4 (7)	

^a^Percentages may not add up to 100% due to rounding.

The telemanagement system showed positive satisfaction responses from the patients for most categories, including simplicity (41/56, 73%), effectiveness (101/112, 90.2%), acceptability (56/56, 100%), usability (110/112, 98.2%), reliability (162/168, 96.4%), level of confidence (55/56, 98%), and ability for remote assessment (38/56, 68%).

## Discussion

### Principal Findings

Our study explores the feasibility of oxygen therapy with noninvasive positive pressure ventilation therapy versus osteopathic manipulative respiratory and physical therapy techniques in COVID-19 patients.

Only 56 of the 60 patients (93%) completed the treatment until recovery and achieved all of the required outcomes. The therapeutic procedures were well tolerated, and the clinical symptoms significantly improved over a relatively short time.

The telemanagement application addressed variance in the clinical outcomes and maximized the benefits from the specialists’ expertise. Telemanagement and telemedicine applications have a wide range of implementations, and there has been robust evidence about their clinical benefits, cost-effectiveness, simplicity, and positive impact on critical care safety and quality [56].

The telemanagement decision support option provided many potential benefits by reducing the specialist’s workload caused by regular revision of all the results; this option allowed patients’ measurement patterns to indicate health status deteriorations and the identification of patients with higher priorities for revision.

Regardless of the broad usage of smart devices in health telemanagement and the collection of data, medical applications of deep learning approaches for making predictions are still considered to be challenging [57]. In this study, depending on the prevailing health conditions, deep learning was used to assist in defining the essential features, predictions, and contextual detections of patterns.

Deep learning was also used as a recognized candidate to predict the probability that a future patient would experience therapeutic failure; this was due to its ability to exploit the intramodality correlations efficiently that would allow for extraction of the hierarchical representations of the data, as well as its ability to perform feature extractions.

The results obtained from this study clearly demonstrated that noninvasive BiPAP was able to significantly improve the clinical status of patients infected with SARS-CoV-2. Patients’ PaO_2_ and oxygen saturation were elevated over a relatively short time. Patients’ respiratory frequency and heart rate decreased. Furthermore, 100% (30/30) of Group A patients did not require any hospitalization or intubation and did not experience any complications.

The results of this study suggest that the application of home-based oxygen BiPAP therapy could improve the respiratory status of patients with COVID-19 pneumonia. It could also significantly improve the arterial blood gas status of the patients without any negative influence on hemodynamics.

It was also found that the application of oxygen with BiPAP at an early stage could reduce the need for intubation along with its related complications. The respiratory complications of COVID-19 can be attributed to a rise in the capillary alveolar membrane permeability, which can lead to pulmonary edema. Thus, noninvasive BiPAP may have contributed to prevention of deterioration by impeding the consequent pulmonary edema via positive pressure [19].

Nevertheless, some side effects of BiPAP were noticed in this study, such as facial skin and eye irritation, mild oropharyngeal dryness, mild abdominal gaseous distention, and stomach pain. Using appropriate face masks with good compatibility, along with avoiding mouth respiration and guiding nasal respiration, have been found to be effective in decreasing these side effects.

Regarding the group treated with osteopathic manipulative respiratory and physical therapy techniques, the improvement in chest CT scans was not significantly different from baseline but seemed to be clinically relevant, while there was a significant improvement in the rest of the outcomes. The clinical symptoms had also improved over a relatively short time. The osteopathic manipulative respiratory techniques that were used with this group have been reported in several studies to have beneficial effects in treating pneumonia [58]. Our main motives for the use of these techniques were focused on immunity improvement, blood clotting prevention, as well as the absence of any noticeable side effect.

Although NIV was found to be more effective, combining the reverse Trendelenburg with prone positioning can be beneficial as well. In our study, it was reported to be tolerable and comfortable by the patients. Some studies investigated the effects of each position [41,42]; one of those studies demonstrated that prone positioning contributed to improvement of the ventilation perfusion mismatch in COVID-19 patients by inducing dorsal lung region recruitment, alveolar shunt reduction, tidal volume, and end-expiratory lung volume improvement [41]. However, we found that prone positioning can induce abdominal push on the diaphragm, especially with patients that have a protruded abdomen, which can limit diaphragmatic excursion. In another study, reverse Trendelenburg positioning by tilting the patient’s head up by 25 degrees showed a decrease in abdominal push on the diaphragm; therefore, an increase in functional residual capacity and lung compliance were observed [42]. Thus, we combined the two positions to avoid any limitations in diaphragmatic movement.

While the group treated with physical therapy showed less-significant results, most of the patients in our study reported relaxation effects immediately after application of cephalic traction and muscle energy techniques for scalene muscles. Similarly, another study demonstrated that these techniques had an effective role in improving vital capacity, increasing respiratory muscle efficiency, increasing cervical flexibility, and decreasing fatigability levels [43]. This study also revealed that the more the lengths of the scalene muscles changed per unit volume, the lower the alveolar pressure became. Thus, the ventilation volume through thoracic expansion increases when the scalene muscles maintain reasonable lengths. Likewise, the rib-raising technique was reported to be relaxing to the patients, which is consistent with another study that found this technique to have an immediate reduction in the activity of the sympathetic nervous system without causing any alteration in parasympathetic activity or in the hypothalamic-pituitary-adrenal axis [44].

The patients’ feelings of arousal that were reported after having intermittent pressure to the suboccipital area can be attributed to the ability of this technique to cause arterial vasomotion at rates usually associated with the cranial rhythmic impulse, as demonstrated in another study [45].

In our study, osteopathic lymphatic thoracic pump techniques with respiratory assist, myofascial release to the thoracic inlet, and the pedal lymphatic pump technique also indicated good tolerability; they were also reported in another study to be advantageous for treating pneumonia by targeting the lymphatic flow, activating autonomic-mediated intrinsic lymphatic contractility, improving respiratory function, and improving circulation. In addition, the thoracic lymphatic pump techniques and thoracic inlet release have been shown to increase chemokines and cytokines in the thoracic vessels as well as in the intestinal lymph vessels, while the pedal lymphatic pump technique improves flow into the lymphatic systems [59]. The outcomes of the lymphatic pump techniques, including improvement in serum interferon levels [46], were carefully directed toward our study sample as we have taken into consideration that COVID-19 induces hyperactivation of the immune system in the severe stages, while it usually induces impairment of the immune system in the early stages. Therefore, immune response suppression may be targeted in the severe stages, while in the early stages, which was the case in our study, reduction of the viral load by stimulating type I interferon should be targeted [60].

It was also observed that in the first group, NIV promoted a significant increase in chest wall volumes directly after application, which could be due to passive expansion. On the other hand, manual diaphragmatic releasing techniques in the second group contributed to positive outcomes of our study by improving the mobility of the chest wall immediately after the intervention, which was in accordance with several studies [49-52].

Compared to physical therapy techniques, BiPAP therapy outcomes were confirmed by this study to be much more effective and promising. Even though physical therapy techniques do not require equipment, those techniques need to be investigated further in order to be considered promising.

Overall, regarding posttreatment satisfaction with our proposed telemanagement health care systems, positive responses were given by most of the patients, even in the group treated with physical therapy, despite longer recovery periods. This was attributed to the advantage of the second group’s costs being lower than those of the first group.

### Limitations

Our study had some limitations. The study included a relatively small number of patients. Thus, further large randomized controlled trials with larger sample sizes are recommended. In addition, osteopathic manipulative respiratory and physical therapy techniques may have a role in elevating recovery rates and improving outcomes of patients with COVID-19. Therefore, further randomized controlled trials with larger sample sizes would be required to determine the therapeutic extent of these techniques.

### Conclusions

From this study, it was found that in the early stages of SARS-CoV-2 pneumonia, home-based oxygen BiPAP ventilation can reduce the need for endotracheal intubation. It can also be an effective prophylactic treatment approach to avoid exacerbation of this disease and the need for hospitalization. Home-based oxygen BiPAP ventilation was more effective than osteopathic manipulative respiratory and physical therapy techniques, as it was associated with shorter recovery periods. A home-based COVID-19 telemanagement system with decision support services showed satisfying outcomes and may be recommended in certain cases as an effective solution for the extreme shortage of hospital beds caused by this pandemic. Further investigations are still required to determine the effectiveness of the osteopathic manipulative respiratory and physical therapy techniques in the management of COVID-19 patients in the early stages.

## Appendix

Multimedia Appendix 1 CONSORT-EHEALTH checklist (V 1.6.1).

## Abbreviations

BiPAP: bilevel positive airway pressure
CT: computed tomography
ICT: information and communication technology
NIV: noninvasive ventilation
